# Volume of hepatoid component and intratumor M2 macrophages predict prognosis in patients with hepatoid adenocarcinoma of the stomach

**DOI:** 10.1007/s10120-024-01562-x

**Published:** 2024-11-03

**Authors:** Yoshiaki Taniguchi, Daisuke Kiyozawa, Kenichi Kohashi, Shinichiro Kawatoko, Takeo Yamamoto, Takehiro Torisu, Tomoharu Yoshizumi, Masafumi Nakamura, Takanari Kitazono, Yoshinao Oda

**Affiliations:** 1 Department of Anatomic Pathology, Graduate School of Medical Sciences, Kyushu University, Maidashi 3-1-1, Higashi-Ku, Fukuoka, 812-8582 Japan; 2 Department of Pathology, Graduate School of Medicine, Osaka Metropolitan University, Osaka, Japan; 3 Department of Medicine and Clinical Science, Graduate School of Medical Sciences, Kyushu University, Fukuoka, Japan; 4 Department of Surgery and Science, Graduate School of Medical Sciences, Kyushu University, Fukuoka, Japan; 5 Department of Surgery and Oncology, Graduate School of Medical Sciences, Kyushu University, Fukuoka, Japan

**Keywords:** HAS, TAMs, CSF-1

## Abstract

**Background:**

Hepatoid adenocarcinoma of the stomach (HAS), a subtype of gastric cancer (GC), includes multiple tumor components, such as enteroblastic and tubular adenocarcinoma components. However, which component mostly contributes to the aggressive behavior of HAS remains unclear. Moreover, the role of tumor-associated macrophages (TAMs) has not been explored in HAS. This study evaluated the clinical significance of the proportion of the hepatoid component within the tumor, CD163 + macrophages, and macrophage colony-stimulating factor-1 (CSF-1) in HAS.

**Methods:**

In total, 56 cases of primary HAS were analyzed. In each case, hepatoid (HC), enteroblastic (EC), and tubular (TC) components were identified, and the ratio of HC to the entire tumor (hepatoid component ratio, HCR) was assessed to examine the correlation between HCR and clinicopathological features. Immunohistochemical staining for CD163 and CSF-1 was performed, and differences in immunohistochemical results among the three tumor components were analyzed. In each tumor component, the prognostic impact of CD163 and CSF-1 was examined.

**Results:**

A high HCR was associated with worse overall survival (OS). CD163 + TAMs and CSF-1 immunoreactivity score in HC were significantly higher than those in the other components. High infiltration of CD163 + TAMs and a high CSF-1 immunoreactivity score in HC were associated with an aggressive course and worse OS. Multivariate analysis revealed the proportion of HC in HAS as an independent prognostic factor (HR = 3.176, *p* = 0.006).

**Conclusions:**

The HCR and CD163 + TAMs may be useful prognostic predictors, and TAMs may be novel therapeutic targets of HAS.

**Supplementary Information:**

The online version contains supplementary material available at 10.1007/s10120-024-01562-x.

## Introduction

Hepatoid adenocarcinoma of the stomach (HAS) is a special subtype of gastric cancer (GC) that is predominantly composed of polygonal cells with plentiful eosinophilic cytoplasm, similar to hepatocellular carcinoma [1, 2]. The incidence of venous invasion and liver metastases in HAS is high, resulting in a significantly poorer prognosis compared to conventional gastric adenocarcinoma [3]. HAS often includes multiple tumor components such as enteroblastic (EC) and tubular adenocarcinoma (TC) components; however, it remains unclear which component mostly contributes to prognosis. Conventional adenocarcinoma is sometimes associated with a focal hepatoid component (HC); such adenocarcinomas were previously regarded as HAS [4]. However, no studies have examined the prognostic impact of a focal HC within the tumor.

In conventional GC, evaluating the tumor microenvironment (TME) is important to predict the efficacy of immune checkpoint inhibitors (ICIs) such as anti-PD-L1 antibody [5]. However, there are few detailed studies on the TME of HAS. Kang et al. reported that the presence of tumor-infiltrating lymphocytes (TILs) suggests that patients with HAS may be appropriate candidates for ICI therapy [6]. However, no studies have compared the TME between the hepatoid and non-hepatoid components of HAS.

In the TME of GC, tumor-associated macrophages (TAMs) have received increasing attention [7]. TAMs can be divided into classically activated M1 macrophages and alternatively activated M2 macrophages, which are involved in tumor growth, metastasis, angiogenesis, and therapy resistance [8]. M2 macrophages reportedly secrete CHI3L1, which promotes metastasis [9]. Therefore, GCs with a high density of TAMs have a poor prognosis [9]. Additionally, GC secretes macrophage colony-stimulating factor-1 (CSF-1), which is a cytokine involved in the differentiation of macrophages into the M2 phenotype [10]. In some cancers, high CSF-1 expression is associated with a poor prognosis, and inhibition of the CSF-1/CSF-1R pathway increases anti-tumor activity [11–14]. Currently, no studies have investigated the clinical significance of M2 macrophage in HAS.

In this study, we evaluated the clinical significance of the HC in GC and compared the number of M2 macrophages between the hepatoid and non-hepatoid components of HAS.

## Materials and methods

### Case selection

We retrospectively reviewed 3,515 surgically resected cases of GC that were registered at the Department of Anatomic Pathology of Kyushu University from 1986 to 2023. Among these, 56 cases of patients with primary HAS, which was defined as GC that included an HC regardless of its volume, were selected. The definition of an HC was based on the World Health Organization (WHO) classification of HAS; predominantly composed of polygonal cells with plentiful eosinophilic cytoplasm, similar to hepatocellular carcinoma [2]. Immunohistochemical expressions of alpha-fetoprotein (AFP), glypican 3, and SALL4 at initial diagnosis were as shown; AFP-positive: 42/56 cases, glypican 3-positive: 44/56 cases, SALL4-positive: 35/56 cases. All 56 patients underwent curative resection without preoperative chemotherapy or radiation therapy. The clinicopathological features of the 56 patients are showed on Table 1.

**Table 1:** Clinicopathological features of selected 56 cases

	*n* = 56
*Age*	
Mean (range)	69.9 (49–87)
*Sex*	
Male	43 (77%)
Female	13 (23%)
*Size*	
Mean (range), cm	5.8 (2.0–16.3)
*Invasion depth*	
pT1-2	17 (30%)
pT3-4	39 (70%)
*Lymphatic permeation*	
(−)	26 (46%)
(+)	30 (54%)
*Vasucular invasion*	
(−)	13 (23%)
(+)	43 (77%)
*Lymph node metastasis*	
(−)	16 (29%)
(+)	40 (71%)
*Liver metastasis*	
(−)	34 (61%)
(+)	22 (39%)
*Tubular component*	
(−)	21 (37%)
(+)	35 (63%)
*Enteroblastic component*	
(−)	41 (73%)
(+)	15 (27%)

### Histological review

**Fig. 1:**
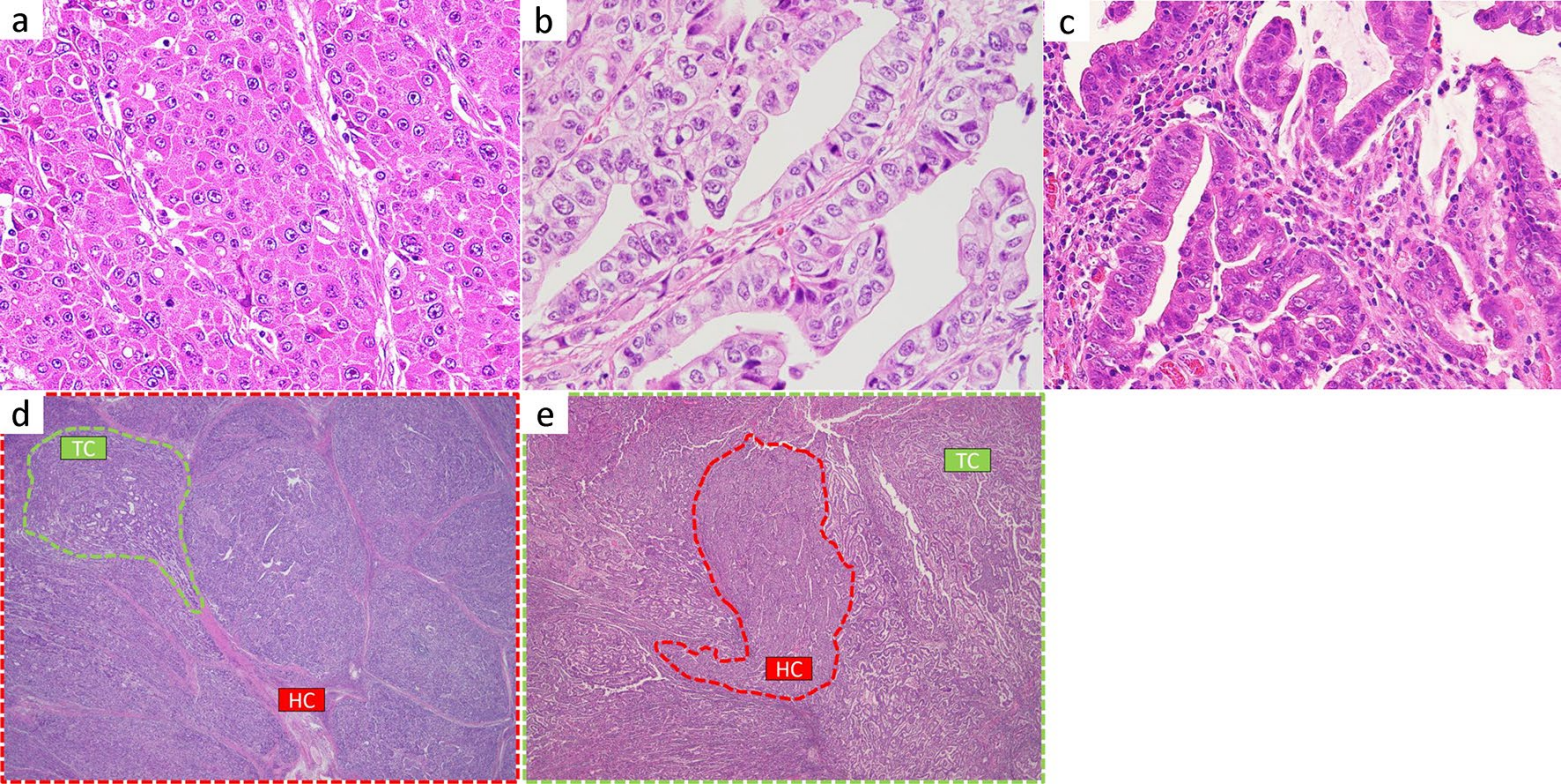
Representative histological images of three tumor components **a** hepatoid component; **b** enteroblastic component; and **c** tubular component), high HCR case (**d**) and low HCR case (**e**)

All 56 cases were histologically reviewed on the largest cutting surface. When the largest cutting surface was not available, we reviewed as many sections as possible. A minimum of 2 and a maximum of 26 sections, averaging 7 sections/case, were reviewed. In each case, tumor components were divided into HC, EC (tumor showing a tubulopapillary architecture composed of columnar cells and clear cytoplasm, resembling early fetal gut epithelium, defined by WHO classification [2]), and TC. The ratio of the HC to the entire tumor (HC ratio, HCR) was grossly assessed (0%–100%, in 10% increments). All 56 cases were divided into high and low HCR groups using the receiver operating characteristic (ROC) curve (high: ≥ 80%, low: < 80%). Representative images of each tumor component, high HCR cases, and low HCR cases are shown in Fig. 1. The correlation between the HCR in each case and clinicopathologic features was examined.

### Immunohistochemical analysis and in situ hybridization

For immunohistochemical analysis, representative formalin-fixed and paraffin-embedded tumor tissues from all cases were sectioned into 3-μm slices. Immunohistochemical staining was performed using the universal immunoperoxidase polymer method (Envision Kit and EnVision Flex Kit; Dako, Tokyo). Antigen retrieval was conducted by heating the slides in 10-mM sodium citrate (pH 6.0) or Target Retrieval Solution (Dako, Carpinteria, CA) or ethylenediaminetetraacetic acid. Initially, we performed molecular subtyping by Epstein-Barr virus (EBV) in situ hybridization and immunohistochemistry (IHC) of mismatch repair (MMR) proteins according to the TCGA molecular classification and our previous study [15–17]. Immunohistochemical staining of CD163, CSF-1, CD8, and AFP were then performed. The antibodies used for IHC are summarized in Supplementary Table 1.

**Fig. 2:**
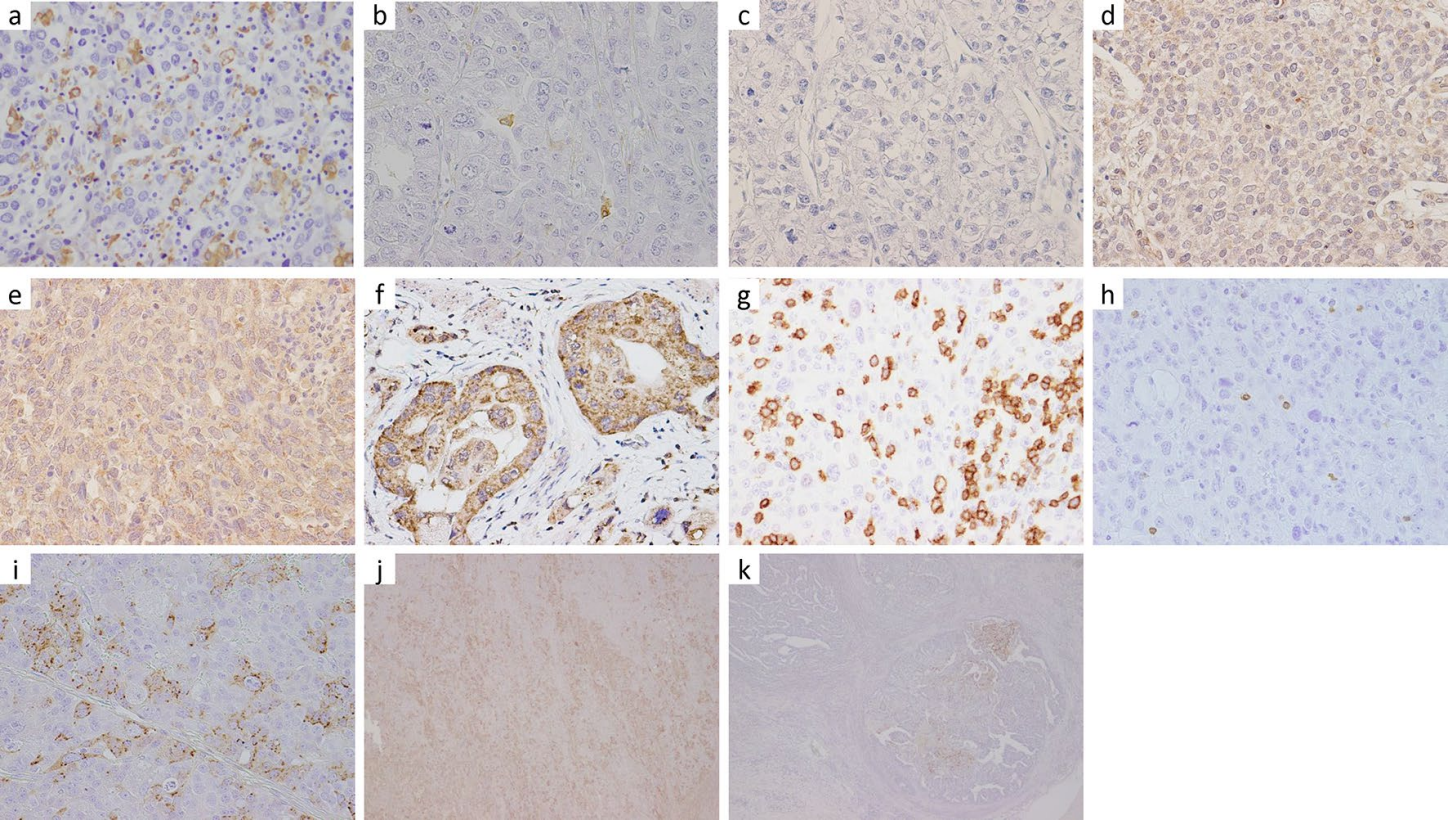
Representative immunohistochemical images of CD163 (**a**, high; **b**, low), CSF-1 (**c**, negative; **d**, weak; **e**, moderate; and **f**, strong), CD8 (**g**, high; **h**, low), AFP (**i**) in the hepatoid component, AFP high expression (**j**), and AFP low expression (**k**)

To analyze M2 macrophage infiltration and TILs, anti-CD163 and anti-CD8 antibodies were selected, respectively [18, 19]. The number of M2 macrophages and CD8 + TILs in each tumor component were assessed at five different high-power fields (HPF) (× 400). The median values (M2 macrophages in HC: 66/HPF, M2 macrophages in EC: 32/HPF, M2 macrophages in TC: 14/HPF, TILs in HC: 60/HPF, TILs in EC: 50/HPF, TILs in TC: 117/HPF) were used as the cutoff values to determine the high and low groups. According to a previous study [14], CSF-1 immunoreactivity was semi-quantitatively evaluated based on the staining intensity and proportion of positive staining based on the following equation: immunoreactive score (IS) = intensity score × proportion score. The intensity score ranged from 0 to 3 (0; negative, 1; weak, 2; moderate, 3; strong), while the proportion score ranged from 0 to 4 (0; 0%–19%, 1; 20%–39%, 2; 40%–59%, 3; 60%–79%, 4; 80%–100%). The final IS ranged from 0 to 12 and was used to divide the specimens into the low (IS ≤ 4) and high (IS > 4) expression groups. The ratio of AFP-positive cells to the entire tumor cells was grossly assessed (0–100%, in 10% increments) in each case. All 56 cases were divided into high and low AFP expression groups using the ROC curve (high: ≥ 20%, low: < 20%). Figure 2 shows the representative images of each IHC stain of HC. All immunohistochemical staining results for each sample were independently evaluated by three pathologists (Y.T., D.K., and Y.O.) who were blinded from the clinical data.

### Statistical analysis

All analyses were performed using the JMP Statistical Discovery Software (version Pro 17; SAS Institute, Cary, NC, the USA). Statistical analyses were performed using Pearson’s χ^2^, Fisher’s exact test, or Wilcoxon’s test. Association between the numbers of M2 macrophages and CSF-1 score was examined using Pearson’s correlation test. Overall survival (OS) was defined as the time from surgery to the time of the last follow-up or death from any cause and was estimated using the Kaplan–Meier method and the log-rank test. Cox proportional hazard regression models were used to perform univariate and multivariate analyses of several factors associated with OS. *P* < 0.05 was considered statistically significant.

## Results

### Histological review

**Fig. 3:**
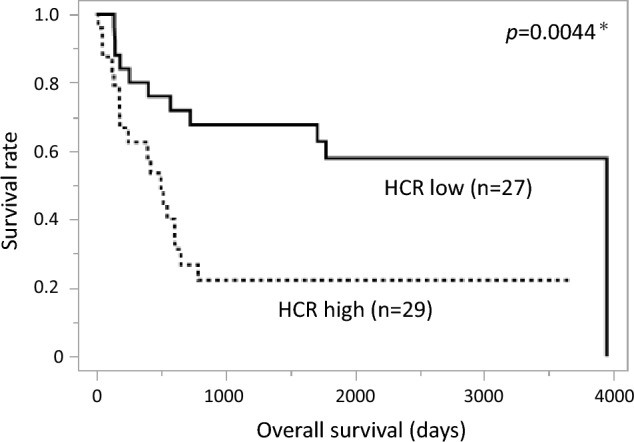
Kaplan–Meier curve of overall survival according to HCR. Patients with a high HCR had a significantly worse OS (*p* = 0.0044) than those with a low HCR

Among the 56 cases of HAS, HC was identified in all cases, TC was identified in 35 cases, and EC was identified in 15 cases. Regarding HCR, the high HCR group (≥ 80%) showed significantly worse OS than the low HCR group (< 80%) (Fig. 3). Additionally, we examined the relationship between HCR and clinicopathological factors and found that the high HCR group had a deeper tumor depth, more frequent liver metastases, and more advanced disease (Table 2a).

**Table 2:** **a** Comparison of clinicopathological features between the high and low HCR groups. **b** Comparison of clinicopathological features between the high and low M2 macrophage groups in HC. **c** Comparison of clinicopathological features between the high and low CSF-1 expression groups in HC

	HCR high	HCR low	p-value
	n = 29	n = 27	
(a)			
*Age*			0.061
Mean (range)	71 (49–84)	75.6 (52–87)	
*Sex*			0.865
Male	22 (76%)	21 (78%)	
Female	7 (24%)	6 (22%)	
*Size*			0.198
Mean (range), cm	6.5 (2.4–16.3)	5.1 (2.0–8.7)	
*Invasion depth*			0.025*
pT1-2	5 (17%)	12 (44%)	
pT3-4	24 (83%)	15 (56%)	
*Lymphatic permeation*			0.432
(−)	12 (41%)	14 (52%)	
(+)	17 (59%)	13 (48%)	
*Vasucular invasion*			0.271
(−)	5 (17%)	8 (30%)	
(+)	24 (83%)	19 (70%)	
*Lymph node metastasis*			0.865
(−)	8 (28%)	8 (30%)	
(+)	21 (72%)	19 (70%)	
*Liver metastasis*			
(−)	14 (48%)	20 (74%)	0.046*
(+)	15 (52%)	7 (26%)	
*Stage*			0.031*
I–II	10 (34%)	17 (63%)	
III–IV	19 (66%)	10 (37%)	

	M2 high in HC	M2 low in HC	*p*-value
	n = 27	n = 29	
(b)			
*Age*			0.950
Mean (range)	71.3 (57–87)	68.6 (49–86)	
*Sex*			0.637
Male	20 (74%)	23 (79%)	
Female	7 (26%)	6 (21%)	
*Size*			0.456
Mean (range), cm	6.3 (2.5–16.3)	5.4 (2.0–12.0)	
*Invasion depth*			0.059
pT1-2	5 (19%)	12 (41%)	
pT3-4	22 (81%)	17 (59%)	
Lymphatic permeation			0.773
(−)	12 (44%)	14 (48%)	
(+)	15 (56%)	15 (52%)	
Vasucular invasion			0.034*
(−)	3 (11%)	10 (34%)	
(+)	24 (89%)	19 (66%)	
*Lymph node metastasis*			0.025*
(−)	4 (15%)	12 (41%)	
(+)	23 (85%)	17 (59%)	
*Liver metastasis*			
(−)	11 (41%)	23 (79%)	0.002*
(+)	16 (59%)	6 (21%)	
*Stage*			0.006*
I–II	8 (30%)	19 (66%)	
III–IV	19 (70%)	10 (34%)	

	CSF-1 high in HC	CSF-1 low in HC	*p*-value
	n = 21	n = 35	
(c)			
*Age*			0.887
Mean (range)	71 (57–84)	70 (49–87)	
*Sex*			0.563
Male	17 (81%)	26 (74%)	
Female	4 (19%)	9 (26%)	
*Size*			0.708
Mean (range), cm	5.7 (2.0–16.0)	5.9 (2.2–16.3)	
*Invasion depth*			0.238
pT1-2	8 (38%)	19 (54%)	
pT3-4	13 (62%)	16 (46%)	
*Lymphatic permeation*			0.035*
(−)	6 (29%)	20 (57%)	
( +)	15 (71%)	15 (43%)	
*Vasucular invasion*			0.934
(−)	5 (24%)	8 (23%)	
( +)	16 (76%)	27 (77%)	
*Lymph node metastasis*			0.212
(−)	4 (19%)	12 (34%)	
( +)	17 (81%)	23 (66%)	
*Liver metastasis*			
(−)	11 (52%)	23 (66%)	0.324
( +)	10 (48%)	12 (34%)	
*Stage*			0.238
I–II	8 (38%)	19 (54%)	
III–IV	13 (62%)	16 (46%)	

*Significant

### Molecular subtyping of HAS according to the TCGA classification

There were no EBV-positive cases, while five cases showed complete loss of MLH1 and PMS2 expressions in all tumor components. In the 51 cases, all MMR proteins were retained. Based on these, we categorized the 56 cases as follows: EBV group (EBER-positive), no cases (0%); MSI group (MLH1 and PMS2 loss), 5 cases (9%); and CIN/GS group (EBER-negative and MSS), 51 cases (91%). Our results are similar to that of a previous study [16].

### Association between the number of M2 macrophages in each tumor component and clinicopathological features

**Fig. 4:**
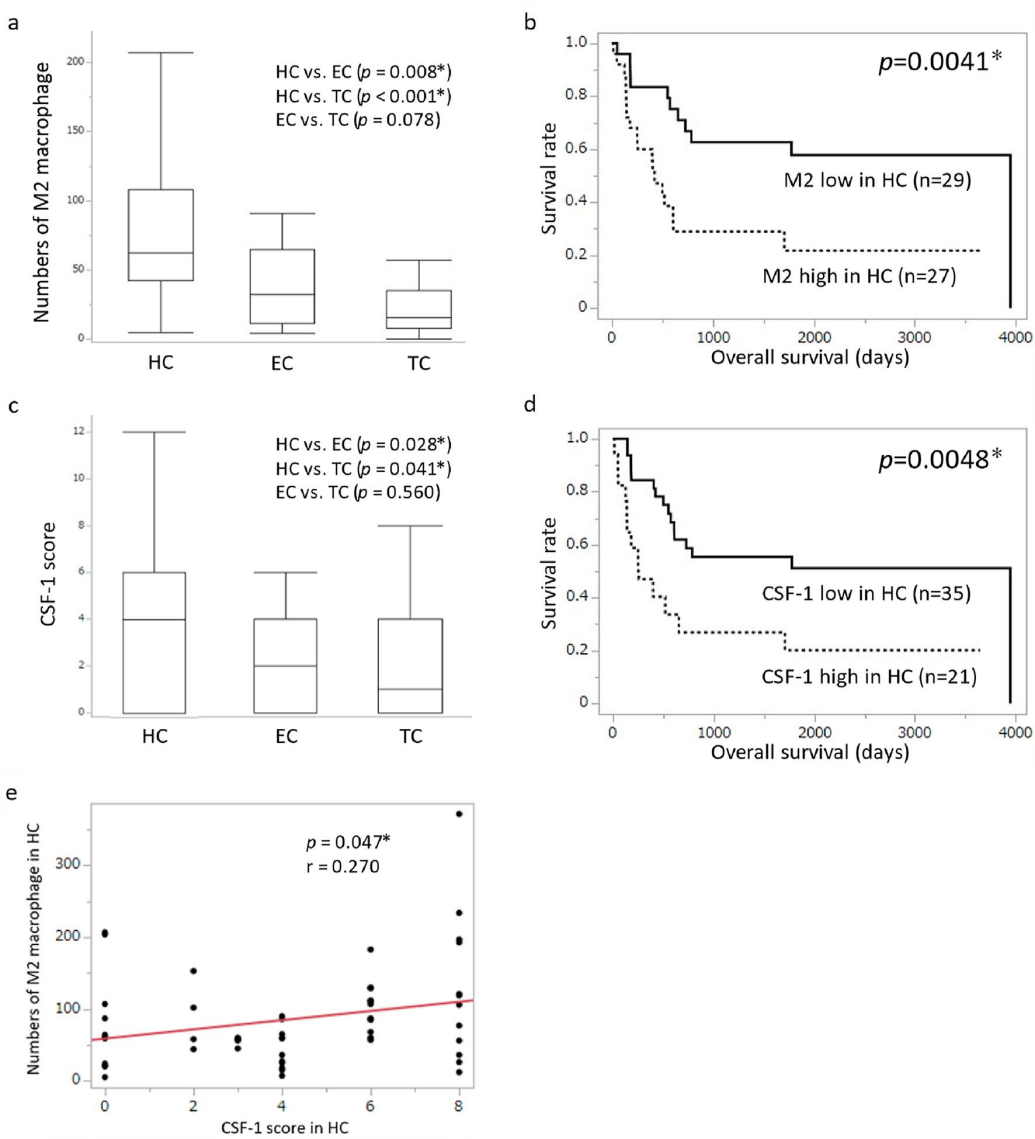
**a** Comparison of number of M2 macrophages among the three tumor components. There were significantly more M2 macrophages in HC than in EC or TC (HC vs EC: *p* = 0.008, HC vs TC: *p* < 0.001) (**a**). Between EC and TC, EC tended to have more M2 macrophages than TC, but the difference was not significant (*p* = 0.078). **b** Kaplan–Meier curve of overall survival according to the number of M2 macrophages in HC. In HC, patients with a high number of M2 macrophages had a significantly worse OS than those with a low number of M2 macrophages (*p* = 0.0041). **c** Comparison of CSF-1 immunoreactivity score among the three tumor components. HC had significantly higher CSF-1 scores than EC or TC (HC vs EC: *p* = 0.028, HC vs TC: *p* = 0.041). Comparing EC and TC, CSF-1 score tended to be higher in EC, but the difference was not significant (*p* = 0.560). **d** Kaplan–Meier curve of overall survival according to the CSF-1 score in HC. In HC, the high CSF-1 expression group had significantly worse OS than the low CSF-1 expression group (*p* = 0.0048). **e** Pearson’s correlation coefficient analysis between the degree of infiltration of M2 macrophages and CSF-1 score in HC. The degree of infiltration of M2 macrophages in HC significantly correlated with the CSF-1 score (*p* = 0.047)

There were significantly more M2 macrophages in HC than in EC or TC (HC vs EC: *p* = 0.008, HC vs TC: *p* < 0.001) (Fig. 4a). Between EC and TC, there tended to be more M2 macrophages in EC than in TC, but the difference was not significant (*p* = 0.078) (Fig. 4a). In each tumor component, we compared the clinicopathological features of M2 macrophages between the high and low groups. In HC, the high M2 macrophage group had a significantly worse OS than the low M2 macrophage group (Fig. 4b). Meanwhile, there was no significant difference in prognosis between EC and TC (Supplementary Fig. 1a-b). In HC, the high M2 macrophage group had a higher frequency of venous invasion, lymph node metastasis, liver metastasis, and more advanced stage than the low M2 macrophage group (Table 2b). In EC, the high M2 macrophage group had a higher proportion of older patients than the low M2 macrophage group (Supplementary Table 2a). In TC, the high M2 macrophage group had a larger tumor size than the low M2 macrophage group (Supplementary Table 2b). These suggest that the degree of infiltration of M2 macrophages in HC, but not in EC or TC, correlated with an aggressive behavior and poor prognosis in HAS.

### Association between CSF-1 status in each tumor component and clinicopathological features

CSF-1 immunoreactivity scores were calculated for each tumor component. HC showed significantly higher CSF-1 scores than EC or TC (HC vs EC: *p* = 0.028, HC vs TC: *p* = 0.041) (Fig. 4c). Between EC and TC, CSF-1 scores tended to be higher in EC than TC, but the difference was not significant (*p* = 0.560) (Fig. 4c). In each tumor component, we compared clinicopathological features between the high and low CSF-1 expression groups. In HC, the high CSF-1 expression group had significantly worse OS (Fig. 4d) and a higher frequency of lymphatic invasion (Table 2c) than the low CSF-1 expression group. Meanwhile, the CSF-1 score in EC and TC did not correlate with prognosis (Supplementary Fig. 2a–b) and any clinicopathological feature (Supplementary Table 3a–b).

### Association between the number of infiltrating M2 macrophages and CSF-1 score in each tumor component

Pearson’s correlation coefficient analysis revealed a significant correlation between the number of infiltrating M2 macrophages and the CSF-1 score in HC (*p* = 0.047) (Fig. 4e) but no in EC and TC (Supplementary Fig. 3a–b).

### Association between the number of CD8 + TILs in each tumor component and clinicopathological features

There was no statistically significant difference in the degree of infiltration of TILs among each tumor component (Supplementary Fig. 4a). In HC and EC, the low TIL group had significantly worse OS than the high TIL group (Supplementary Fig. 4b–c). Additionally, the degree of infiltration of TILs in TC did not correlate with prognosis (Supplementary Fig. 4d). In HC, the low TIL group had a higher frequency of vascular invasion, lymph node metastasis, and liver metastasis, and had more advanced disease (Supplementary Table 4a). In EC and TC, the low TIL group had a higher frequency of liver metastasis (Supplementary Table 4b–c).

### Association between AFP expression and clinicopathological features

We assessed AFP expression in each case, and compared clinicopathological features between the high and low AFP expression groups. The high AFP expression group tended to have worse OS than the low AFP expression group, but the difference was not significant (*p* = 0.085) (Supplementary Fig. 5a). In high AFP expression group, there were significantly less TILs than in AFP low expression group (Supplementary Fig. 5b). There was no statistically significant difference in the degree of infiltration of M2 macrophages between AFP high and low groups (Supplementary Fig. 5c). The low AFP expression group had a larger tumor size than the high AFP expression group (Supplementary Table 5). There were more AFP-positive cells in the high HCR group than in the low HCR group (Supplementary Fig. 5d).

### Univariate and multivariate analysis

Univariate analysis revealed a significant association between a worse OS and pathological tumor depth, pathological stage, vascular invasion, number of M2 macrophages and TILs in HC, CSF-1 expression in HC, and HCR. Meanwhile, multivariate analysis revealed that HCR had a significant effect on OS (HR = 3.176, *p* = 0.006) (Table 3).

**Table 3:** Univariate analysis and multivariate analysis of various prognostic factors of OS in all cases

Prognostic factors	Univariate			Multivariate		
	HR	95% CI	*P* value	HR	95% CI	*P* value
Age (year)	1.906	0.227–1.207	0.116			
> 73 vs. ≤ 73						
Tumor size (mm)	1.503	0.687–3.286	0.317			
> 39 vs. ≤ 39						
Pathological tumor depth	2.892	1.092–7.658	0.032*	1.448	0.417–5.028	0.559
pT3-4 vs. pT1b-2						
Pathological node stage	2.123	0.854–5.279	0.084			
pN1-3 vs. pN0						
Pathological Stage	3.679	1.621–8.354	0.001*	1.289	0.408–4.072	0.665
III + IV vs. I + II						
Lymphatic invasion	1.416	0.662–3.032	0.370			
Present vs. absent						
Vascular invasion	5.925	1.399–25.087	0.008*	3.863	0.880–16.959	0.073
Present vs. absent						
Number of M2 in HC	2.898	1.306–6.432	0.008*	1.656	0.649–4.227	0.289
High vs. low						
CSF-1 expression in HC	2.642	1.229–5.681	0.012*	2.085	0.883–4.923	0.093
High vs. low						
Number of CD8 + TILs in HC	0.363	0.161–0.816	0.014*	0.441	0.184–1.060	0.068
High vs. low						
AFP expression	1.901	0.902–4.005	0.091			
High vs. low						
HCR	2.858	1.290–6.331	0.009*	3.176	1.383–7.294	0.006*
High vs. low						

*Significant

## Discussion

This study showed the clinical significance of HCR and intratumoral M2 macrophages in HAS. A high HCR correlated with tumor depth, liver metastasis, advanced stage, and poor prognosis. Multivariate analysis showed that high HCR within the tumor was an independent prognostic factor of poor prognosis. HC contained more M2 macrophages and showed higher CSF-1 score than the other two (EC and TC) tumor components. M2 macrophages in HC correlated with vascular invasion, lymph node metastasis, liver metastasis, advanced stage and poor prognosis. High CSF-1 score in HC was correlated with lymphatic invasion and poor prognosis. The numbers of infiltrating M2 macrophages significantly correlated with CSF-1 score in HC.

To the best of our knowledge, this is the first report showing that the volume of the HC was an independent prognostic factor in patients with HAS. The possible mechanism of association between poor prognosis and a high HCR may be the immunosuppressive function of AFP produced by HAS [4, 20]. Based on our study, the HCR could be useful for predicting the prognosis of patients with HAS.

Several reports have revealed that TAMs and CSF-1 are correlated with advanced disease or distant metastasis in conventional GC and cancers of other organs [9, 11–14]; however, no reports have examined the clinical significance of TAMs and CSF-1 in HAS. In our study, abundant infiltrating M2 macrophages and a high CSF-1 score in HC were associated with a worse prognosis. This is also the first study to evaluate the clinical significance of M2 macrophages and CSF-1 in HAS. In the TME, CSF-1 secreted by tumor cells binds to CSF-1R expressed on the surface of macrophages, promoting their differentiation into the M2 phenotype, which releases cytokines such as chemokines, inflammatory factors, and growth factors that act in a tumor-promoting manner [8] and may be associated with poor clinical outcomes. Therefore, inhibition of the CSF-1/CSF-1R pathway, may be useful in the treatment of HAS.

HAS with less infiltration of TILs had a poor prognosis, and ICI may be a potential therapeutic strategy [6]. Our study also showed that less infiltration of TILs in HC had significantly worse OS (Supplementary Fig. 4b), which supports previous results. Compared with a previous study [6], we investigated TILs in each tumor component separately (HC, EC, TC), which may provide more accurate information about tumor immuno-microenvironment in HAS.

There was a previous report showing that serum AFP level is an important prognostic indicator in HAS [4], however, there is no report which examine the association between AFP immunohistochemistry and TME, clinicopathological features, and prognosis in HAS. In this study, cases with a high expression of AFP showed significantly less infiltration of TILs in HC. The high AFP expression group tended to have worse OS than the low AFP expression group. These results may suggest that AFP plays an important role in T-cell immunosuppression. Previous report also supported our results [20].

This study had several limitations in addition to its retrospective nature and small-sized cohort. First, the number of cases that had an enteroblastic or tubular component was relatively small. Second, the MSI status was assessed only by immunohistochemical staining and not by genetic testing, which may not accurately assess the MSI status by tumor component.

## Conclusions

The volume of the HC was an independent prognostic factor of HAS. M2 macrophage infiltration and a high CSF-1 score in HC were associated with a worse OS, suggesting that the CSF-1/CSF-1R pathway may be a potential therapeutic target in patients with HAS.

## Supplementary Information

Below is the link to the electronic supplementary material.

Supplementary file1 (PPTX 1386 KB)

Supplementary file2 (XLSX 41 KB)
