# Supplementary material for: Volume of hepatoid component and intratumor M2 macrophages predict prognosis in patients with hepatoid adenocarcinoma of the stomach
Source: Gastric Cancer. 2024 Nov 3;28(1):41–50. doi: 10.1007/s10120-024-01562-x (PMC11706836; doi:10.1007/s10120-024-01562-x)
Supplement: Supplementary file 1 — Supplementary file1 (PPTX 1386 KB) [file 10120_2024_1562_MOESM1_ESM.pptx]

## Slide 1
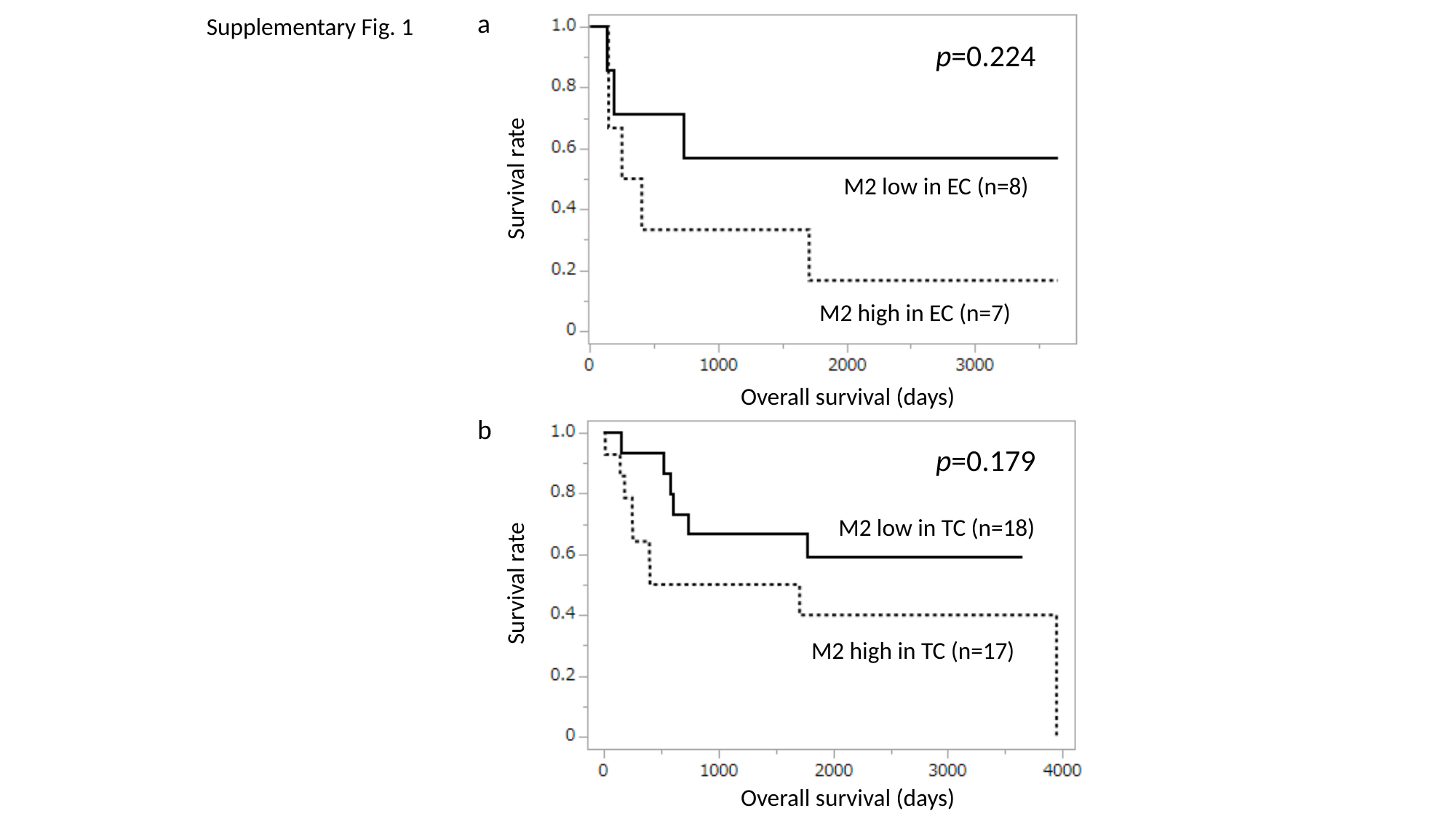

Supplementary Fig. 1
a
p=0.224
Survival rate
M2 low in EC (n=8)
M2 high in EC (n=7)
Overall survival (days)
b
p=0.179
M2 low in TC (n=18)
Survival rate
M2 high in TC (n=17)
Overall survival (days)

## Slide 2
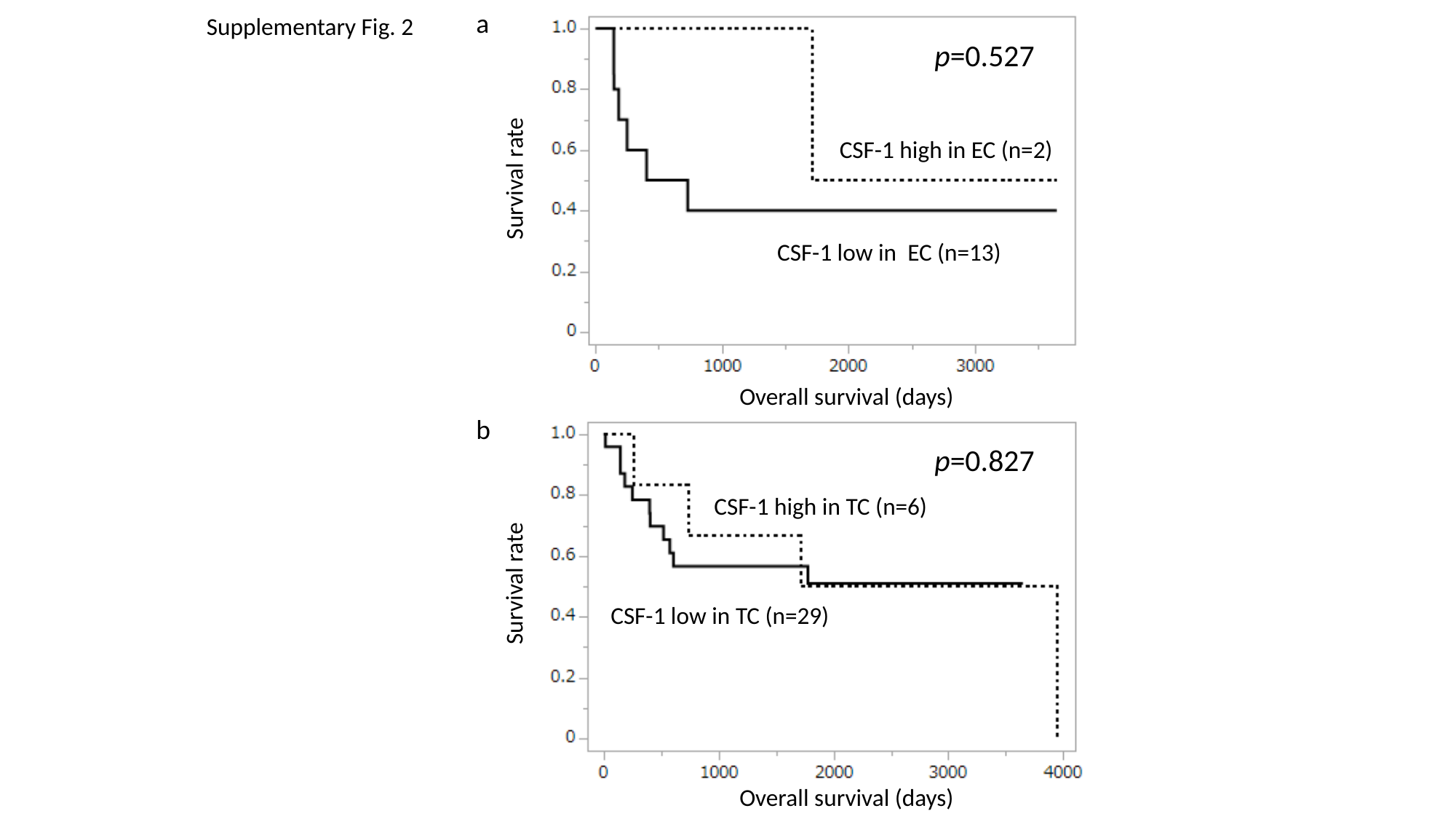

Supplementary Fig. 2
a
p=0.527
CSF-1 high in EC (n=2)
Survival rate
CSF-1 low in EC (n=13)
Overall survival (days)
b
p=0.827
CSF-1 high in TC (n=6)
Survival rate
CSF-1 low in TC (n=29)
Overall survival (days)

## Slide 3
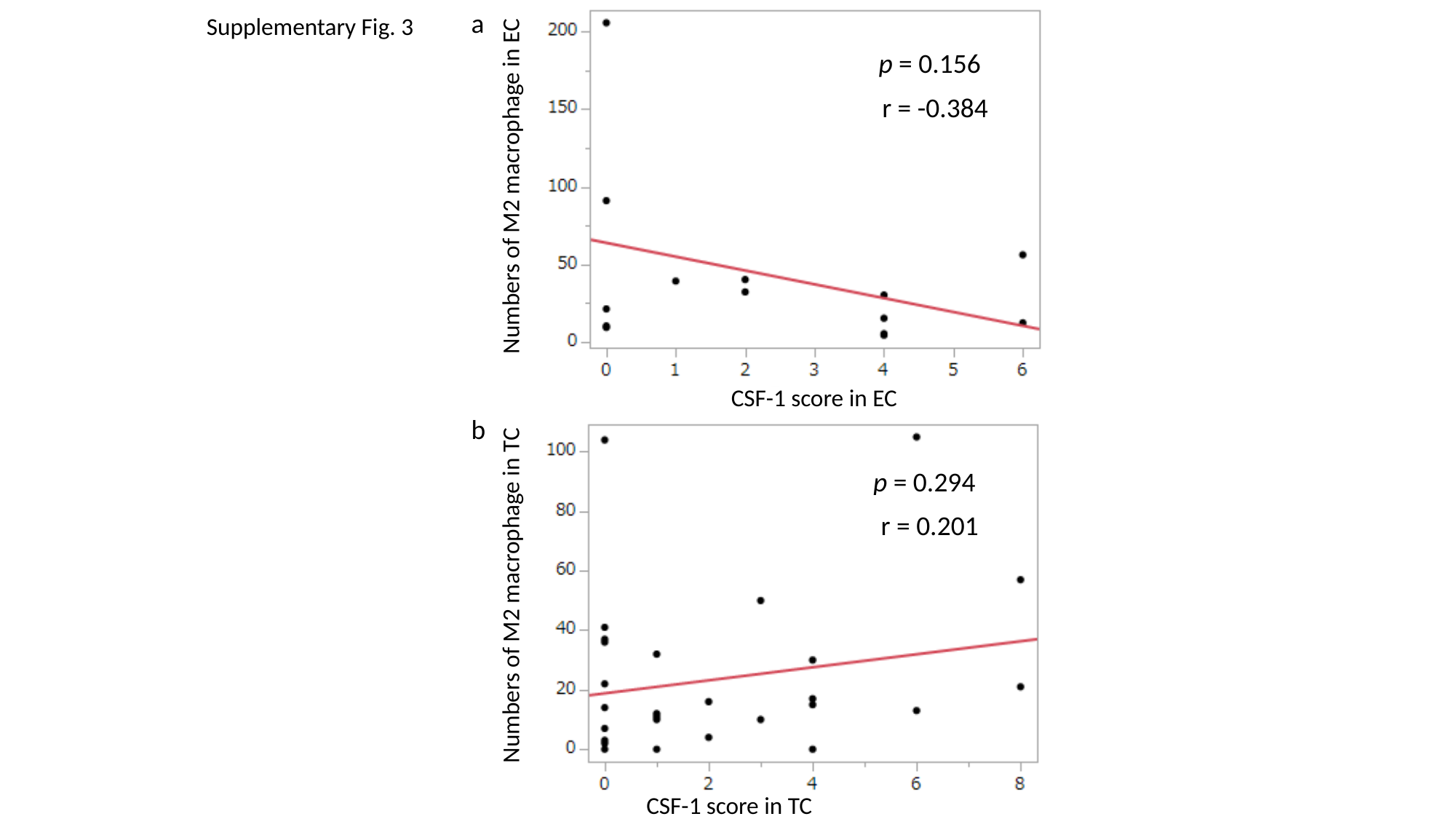

Supplementary Fig. 3
a
p = 0.156
r = -0.384
Numbers of M2 macrophage in EC
CSF-1 score in EC
b
p = 0.294
r = 0.201
Numbers of M2 macrophage in TC
CSF-1 score in TC

## Slide 4
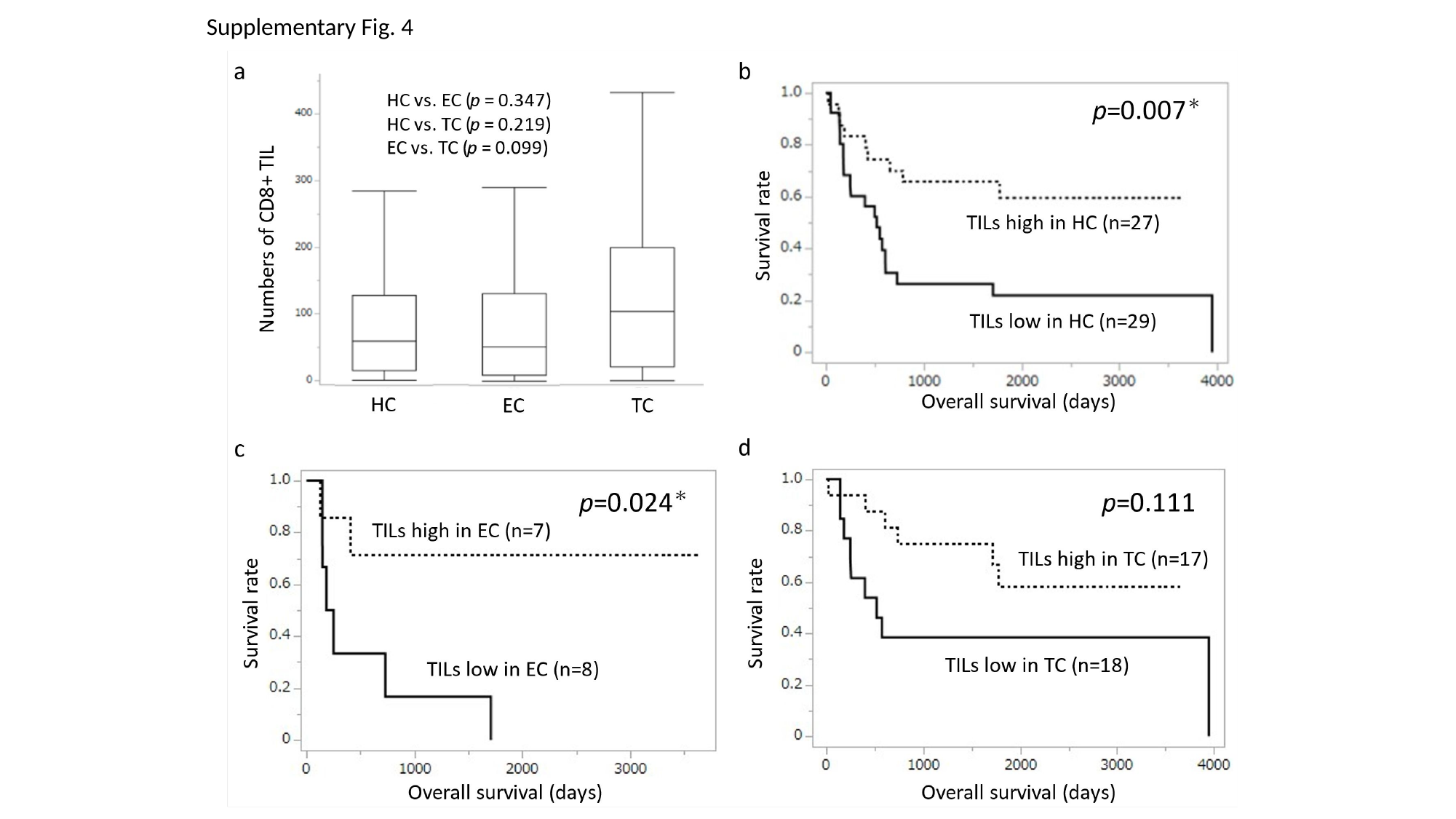

Supplementary Fig. 4

## Slide 5
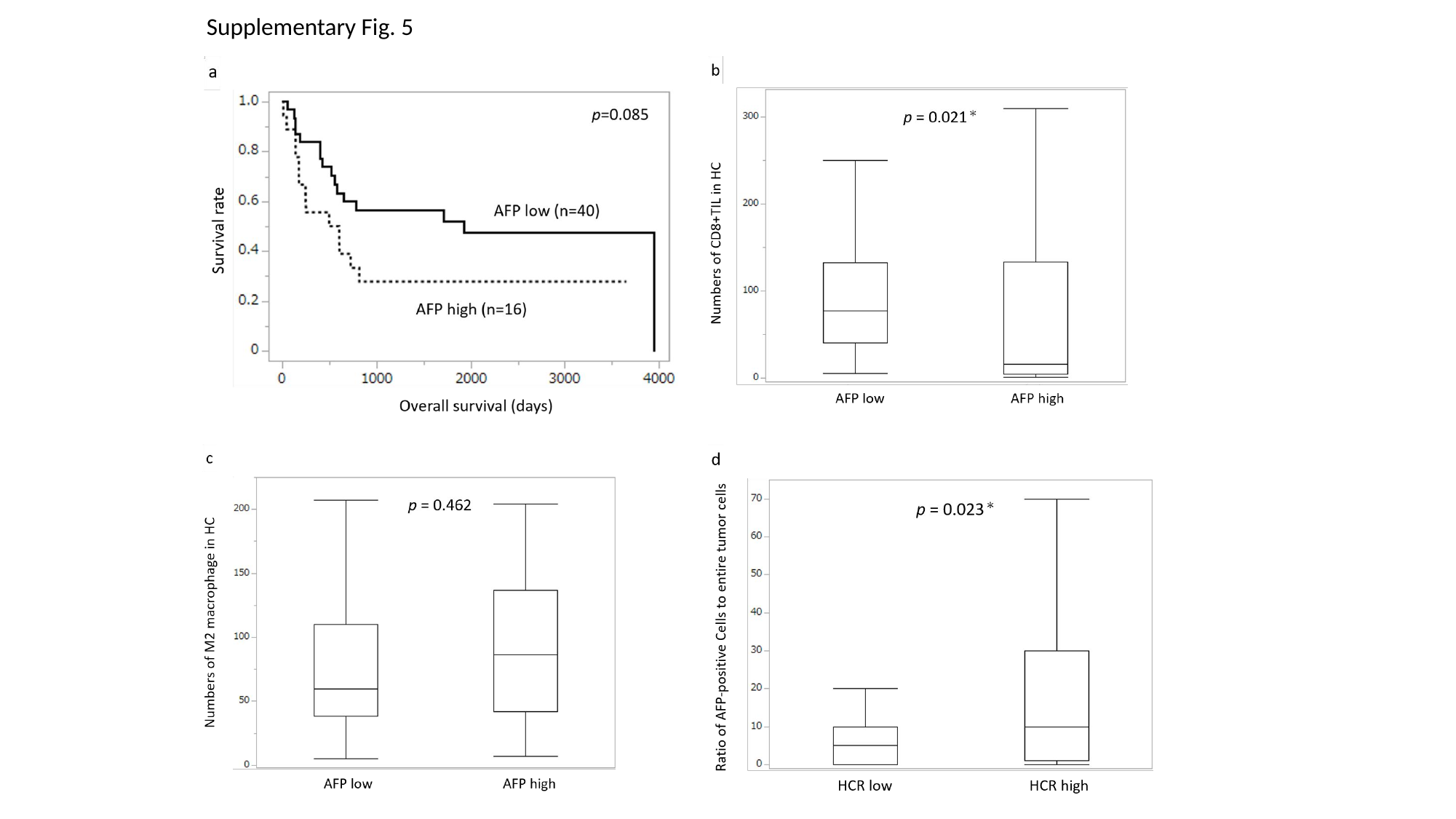

Supplementary Fig. 5
